# Care Coordination for Vulnerable Families in the Sydney Local Health District: What Works for Whom, under What Circumstances, and Why?

**DOI:** 10.5334/ijic.5437

**Published:** 2020-12-02

**Authors:** John G. Eastwood, Suzannah Dewhurst, Sally Hansen, Elaine Tennant, Erin Miller, Maja Lindegaard Moensted, Penelope Fotheringham, Denise De Souza

**Affiliations:** 1 School of Women’s and Children’s Health, The University of New South Wales, Sydney, NSW, AU; 2 Ingham Institute of Applied Medical Research, Liverpool, NSW, AU; 3 Charles Perkins Centre, Menzies Centre for Health Policy, Discipline of Child and Adolescent Health, and School of Public Health, University of Sydney, Sydney, NSW, AU; 4 Sydney Institute for Women, Children and their Families, Camperdown, NSW, AU; 5 Community Health Services, Sydney Local Health District, Camperdown, NSW, AU; 6 Drug Health Services, Sydney Local Health District, Camperdown, NSW, AU; 7 Centre for Research in Education (CRED), Torrens University Australia, Melbourne, VIC, AU

**Keywords:** integrated care, critical realism, care coordination, disadvantaged families, evaluation

## Abstract

**Introduction:**

Healthy Homes and Neighbourhoods (HHAN), an integrated care programme in the Sydney Local Health District (SLHD), seeks to address the needs of disadvantaged families through care coordination, as one of its components. This research aims to determine for whom, when and why the care coordination component of HHAN works, and establish the reported outcomes for clients, service-providers and partner organisations.

**Methods:**

Critical realist methodology was utilised to undertake a qualitative evaluation of the impact of care coordination. Purposive sampling was used to select a total of 37 participants for interview, including consumers, service-providers and key stakeholders. Thematic analysis was undertaken to derive the major modes of intervention of HHAN, and data representing these elements was coded and summarised under contexts, mechanisms and outcomes.

**Results:**

Analysis indicates that care coordination has a positive impact on clients’ sense of independence, self-awareness and outlook on life. Trust and favourable interpersonal relations were identified as major underlying mechanisms for a successful client-provider working relationship. The identified modes of intervention facilitating positive consumer outcomes included accessibility, flexibility and service navigation. Persistent siloes in health and systemic resistance to collaboration was seen to hinder effective care delivery.

**Conclusions:**

This study suggests that a care coordination model may be effective in engaging disadvantaged families in healthcare, assist them in navigating the health system and can lead to beneficial health and social outcomes. Successful implementation of care coordination requires flexible programme design and experienced and skilful clinicians to fulfil the care coordinator role. There is a need to appreciate the negative impact that the complex and siloed health system can have on disadvantaged families.

## Introduction

Individuals experiencing severe disadvantage such as poverty, unstable housing and unemployment often also face adverse social, mental and physical health issues [1,2]. Such factors combined are likely to contribute to intergenerational disadvantage and poor health outcomes [3,4]. Social disadvantage is further compounded by the complexity of the health system, service fragmentation and widespread distrust in systems of authority within many disadvantaged communities [5,6]. As health inequalities for individuals living in pervasive social disadvantage continue to grow [7], more focus is paid on developing service initiatives able to address the complex nature of social exclusion [8]. Integrated care and care coordination have been promoted as a potential answer to the health crisis dominating many disadvantaged communities [9].

Social disadvantage in a health context refers to an increased risk of experiencing adverse health outcomes due to the aforementioned risk factors, also known as social determinants of health [10,11]. Historical social and economic disparities that arise from these determinants often manifest in subpopulations of society, in which the determinants of health work in combination, or independently, contributing to the cycle of disadvantage. Such areas of social disadvantage are evidenced in the Sydney Local Health District (SLHD), where high proportions of residents in suburbs of Redfern, Glebe, Waterloo and Riverwood reside in social housing. These areas have an unemployment rate higher than 20%, compared to less than 3% unemployment in more affluent areas in Sydney [12].

In recognition of the multi-layered nature of disadvantage, and the complexity of the family unit, traditional and acute models of care are increasingly seen as inadequate as the sole methods of delivering healthcare to disadvantaged families [13,14]. Attempting to fill the gaps in service delivery, New South Wales (NSW) Health has declared a commitment to coordinated and integrated models of care [15]. Integrated care can be described as the “provision of seamless, effective and efficient care that reflects the whole of a person’s health needs (…) in partnership with the individual, their carers and family” [11].

Care coordination aims to facilitate this form of healthcare delivery by linking clients to appropriate services, which may also facilitate more systemic connectivity between health care providers [16].

In 2015 SLHD implemented an integrated care initiative for disadvantaged families in the Inner West region of Sydney, Australia. The initiative, known as Healthy Homes and Neighbourhoods (HHAN), is designed as a cross-agency care coordination network for disadvantaged families [17]. The HHAN programme supports families with at least one child under 17 years of age living within SLHD, where the parents or carers have complex health and social care needs. HHAN seeks to provide multiagency wrap-around care through care coordination at a client level, and service integration at a systemic level [18]. The programme operates through both practitioner home-visiting and place-based settings, with the place-based clinicians operating out of a multiagency hub in Redfern and a community centre in Riverwood.

HHAN consists of five service-providers: three senior clinical nurse consultants and two senior social workers. Two service-providers are based in the place-based multiagency hub in Redfern, two service-providers are based in the community centre in Riverwood, and one service-provider covers the families enrolled in HHAN who are located between the two areas. All service-providers work independently, however, come together weekly for case reviews, intake meetings, and business meetings. The historical and current context of HHAN and a more in-depth programme description have been reported elsewhere [19].

Despite long-standing Government commitment to integrated care and support for disadvantaged families in Australia, the evaluations of such programmes are limited [20,21]. Complex interventions such as HHAN contain several interacting components, a broad range of outcomes, and an inexplicit level of subjectivity by those implementing and receiving them, all of which act interdependently of each other [22]. Additionally, whilst a client-centred approach is key to the success of a programme such as HHAN, such an approach poses issues for evaluation, as each family’s wellbeing will improve in different areas over a unique timeframe, making it difficult to relate outcomes to initial aims.

Seeking to overcome these issues, this study employs a critical realist evaluation approach. Such an approach provides a useful framework to identify factors that facilitate or hinder the implementation and outcomes achieved by HHAN in the SLHD context, as well as insights into how findings might be transferred across settings and populations [22]. The aim of this evaluation is to understand what mechanisms of HHAN care coordination work for whom, under what circumstances, and why, and to establish the recorded outcomes for clients, clinicians, and partner organisations.

The Logic Model for HHAN shown in Table 1 illustrates the overall design of the programme, including contextual information, identified interventions, programme mechanisms and change in previously identified causal mechanisms of poor maternal wellbeing (outcomes) [23]. It illustrates the many levels of the health sector targeted by HHAN, including both clinical and non-clinical components. This paper will focus on component two, care coordination, and explore which aspects of the care coordination model have an impact on clients, as well as the wider health environment in which this occurs.

**Table 1:**
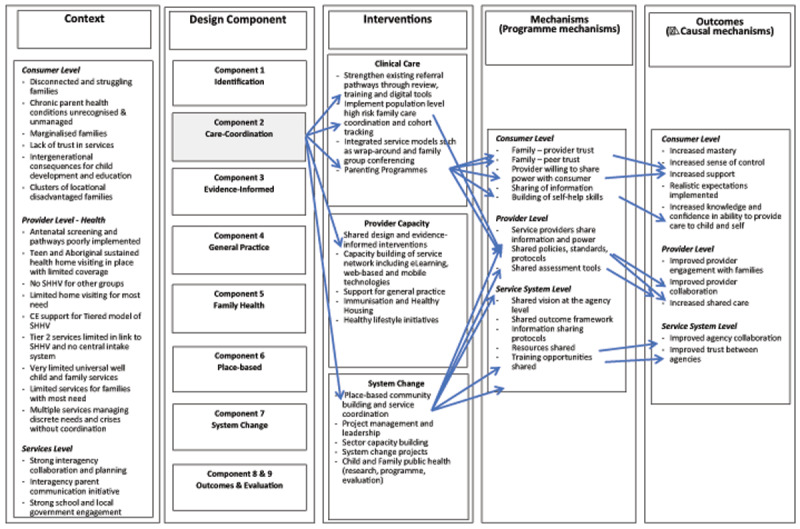
HHAN Logic Model (created 2015).

## Theory and Methods

### Introduction

Realist evaluation methodology acknowledges that programmes work differently in different contexts, and is an effective method of analysing the impact of a social programme in which data can only be drawn from those to whom it has been offered. Realist theory focusses on understanding how programmes generate outcomes, paying attention to the shaping of causal mechanisms by social and economic contexts [24,25]. It employs an analytical unit known as a context-mechanism-outcome (CMO) configuration to understand the interaction between a programme’s contextual setting, the mechanisms evident, and any impacts on client and system outcomes. It is therefore suitable for examining the HHAN programme, as it considers the influence different locations and participants may have on the overall result [25].

**Figure 1:**
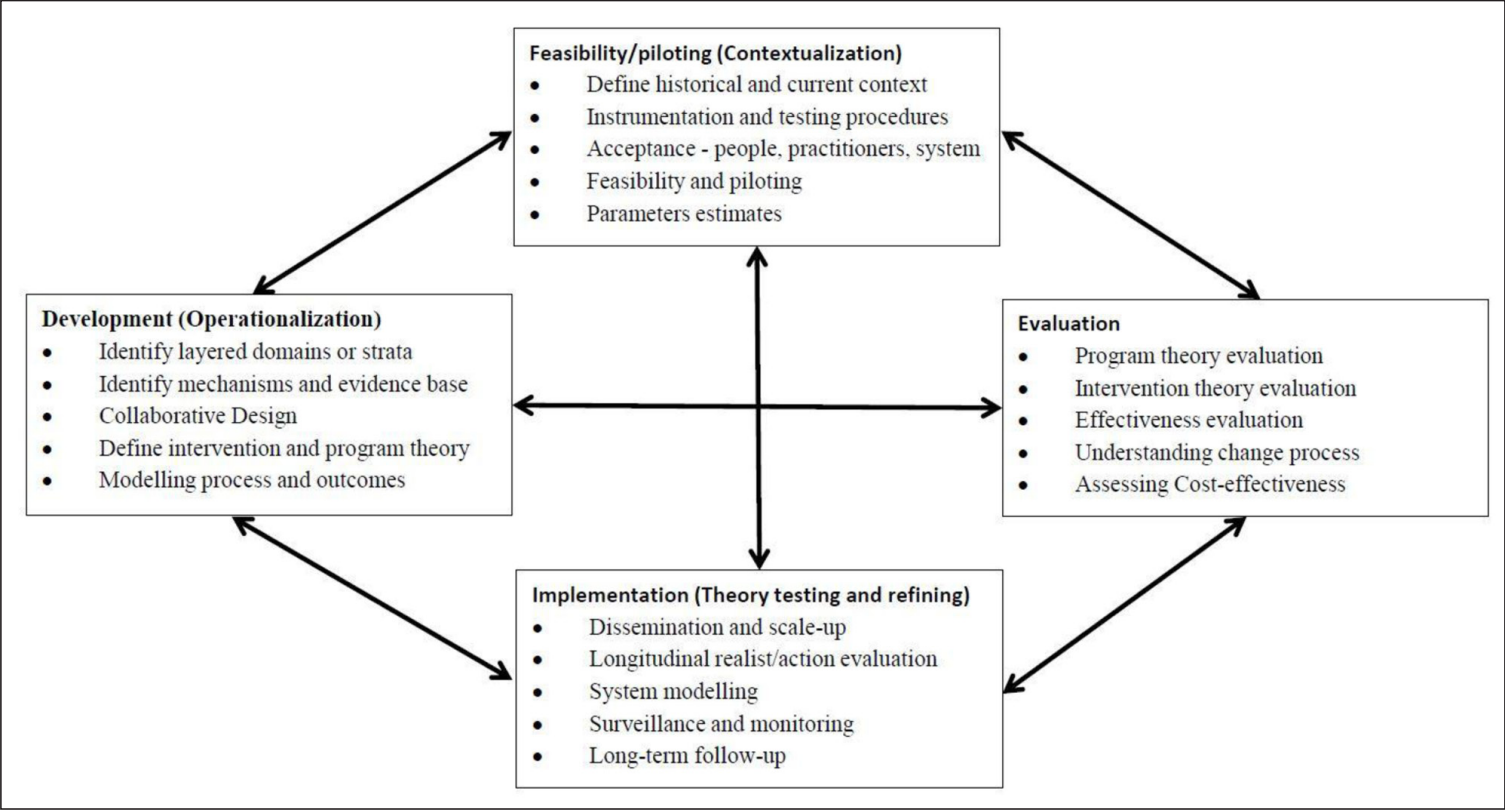
Key elements of the adapted development and evaluation process.

The study methods were informed by the UK Medical Research Council (MRC) Framework with its four components, namely 1) development, 2) feasibility/piloting, 3) evaluation and 4) implementation [26]. This framework was adapted (see Figure 1) and has been previously reported [27,28].

According to the MRC Framework, a key aim of the feasibility and piloting phase is to test the intervention for acceptability among key stakeholders [26]. In the current study, this included HHAN staff, stakeholders from partner organisations and consumers.

### Feasibility/piloting (Contextualisation)

The study was part of the Confirmatory Phase of “Explanatory Theory Building”. Explanatory Theory Building Methods adopt induction, abduction, retroduction and deduction as the central forms of reasoning to develop realist hypotheses based on operationalised theoretical propositions in concrete settings [27]. The critical realist case-study approach to the HHAN pilot built on the evaluation protocols described in earlier papers in this collection[28], and consisted of three phases:

1. Theory and proposition development
2. Observations through qualitative data collection
3. Analysis of CMO configurations.

The initial programme theory for HHAN (see Table 1) has been described in detail elsewhere[19]. Programme propositions are expressed in realist terms as context-mechanism and outcome (CMO) conjectures, including the contextual levels of Self, Situated Activity, Intermediate Level and Macro Level, as proposed by Layder [29].

### Methodological Approach

#### Qualitative Data

For the realist evaluation, qualitative semi-structured interviews with clients, service-providers and key stakeholders were undertaken. Data were collected for an overall evaluation of how the theorised HHAN programme mechanisms manifested in concrete situations. This paper reports on a single aspect of the HHAN logic model, namely the care coordination component. Such a restricted focus allows for a detailed study of the programme theory mechanisms pertaining to the selected component.

Purposive sampling was used for the identification and selection of participants able to provide ‘rich’ and relevant information on the programme [30]. Thirty-seven interviews were conducted with clients of HHAN (n = 15), service-providers (n = 5), the programme manager (n = 1), and relevant stakeholders (n = 16). Stakeholders included individuals who worked with partner organisations such as Health services, Housing services, or financial services, and were involved with HHAN through shared clientele or as part of the steering committee.

Clients were initially invited to participate by their key worker, after which the researchers contacted them directly. Interviews ranged from 20-50 minutes in length and were conducted at a convenient and comfortable location for the clients including at participants’ home, the place-based hub, one in a client’s car, and one via phone call. Clients received a $50 grocery voucher as a thank-you for their participation. Written informed consent was obtained by all participants prior to interviews.

Interviews were undertaken between December 2015 and September 2017. A questioning framework was developed and refined through a three-step realist CMO approach, specifically theory gleaning, theory refining and theory consolidation [31]. This involved firstly obtaining information from users of the programme to gather first-order theories identifying contextual circumstances. As interviews progressed, questions were tailored to refine specific CMO theories. As such, although similar in topic, interview questions were revised and altered throughout the data generation phase as theories were refined and advanced. Overall, clients were asked about their background, reason for referral (context), experiences with the programme and possible outcomes. Service-providers and stakeholders were asked about their experience with the programme, the strengths and weaknesses of the model of care as well as client and service outcomes.

#### Analysis and identification of refined theory

Interviews were audio-taped, transcribed verbatim, de-identified and coded thematically in NVivo v10 software (QSR). Critical realism acknowledges the stratified nature of social reality, and in this study, three levels of social reality were analysed: the programme outcomes at the consumer level, provider level and service system level [32]. Once the 37 interviews were analysed, the team of coders agreed that saturation of themes was attained and theoretical completeness achieved [33]. Thematic analysis was used to deduce patterns and modes of intervention evident in the transcripts. A process of familiarisation, coding, theme development, defining themes and reporting was used to examine CMO configurations emerging in the data. Through an iterative process, each transcript initially underwent line-by-line coding to identify key modes of intervention emerging. Codes were then grouped under higher-order categories through which final mechanisms and contextual factors emerged [34].

To determine CMO configurations, a process of theory gleaning, refining and consolidating was employed [31]. The coded data were categorized according to context (C), mechanism (M) and outcome (O). Next, the various theories were grouped into higher and lower order codes. Theories were then organised connecting the outcomes with identified mechanisms that were triggered to generate those outcomes, and finally the contexts within which those mechanisms were triggered. The analysis was informed by both inductive and retroductive modes of reasoning. This was an iterative process undertaken by the second author. Codes were cross-analysed by a second coder in the same team. The theories were organised and adapted from the layering of reality proposed in the Logic Model, namely: consumer level, provider level and service system level. Consistent theories were then consolidated through team discussion and a final review of the interviews [25].

### Ethics

Written informed consent was obtained before each interview with participants reassured of their anonymity. The study was approved by the Research Ethics and Governance Office, Royal Prince Alfred Hospital, Sydney Local Health District (X15-0138 & HREC/15/RPAH/190).

## Results

### Main findings

The research identified process mechanisms operating within HHAN situated at different levels. At the client level, whole of family care was identified as the key mode of intervention (Table 2). At the provider level, four main mechanisms emerged; accessibility, accompaniment, clinician autonomy and service navigation (Table 3). Service collaboration emerged as the main process mechanism at service delivery level (Table 4). Both positive and negative outcomes were identified at the provider and service delivery levels. Two overarching relational mechanisms, trust and favourable inter-personal relations, emerged as overarching mechanisms required to enable effective consumer engagement.

**Table 2:** CMO configuration for whole of family care.

Characteristic Of Programme	Context	Mechanism	Outcome

Whole of family approach	Client characteristics: – Distrust in healthcare services – Intergenerational trauma* – Financial stress* – Housing and security issues* – Mental and physical health conditions* – Negative social and family experiences*	**Acknowledging** all extended family dynamics and needs	**Awareness** of all extended family dynamics and needs Rapport building with all family members Client supported in the community: – Client independence – Client outlook improved Acknowledgment and acceptance of HHAN by clients, families and community

* Encompassed in the single term ‘vulnerability’ from this point forward.

**Table 3:** CMO configurations for accessibility.

Characteristic of Programme	Context	Mechanism	Outcome

Accessibility	Client characteristics: – Vulnerability – Distrust of health services Programme characteristics: – Home visiting – Place-based initiatives Health system characteristics: – Inflexibility	**Approachable** through multiple modes of communication	Available Service engagement
Accompaniment/intensive hand holding	Client characteristics: – Vulnerability – Disconnected from health services – Fluctuating intensity of client need Programme characteristics: – Programme flexibility – Providers taking dual roles	**Reliability and Persistency**	Responding to changes in client needs Motivation of clients belief in own capability (positive perception) Client independence or Client reliant on providers Risk of case management model
Clinician autonomy	Client characteristics: – Vulnerability – Diversity of age and social, cultural and health background Programme characteristics: – Flexibility	**Confident clinicians**	Clinician holds responsibility for personal case load Clinician and client shared decision making Positive outcomes: – Client independence – Service engagement Negative outcomes: – Staff burnout – Service delivery dependent on experience of staff
Navigation of health system	Health system characteristics: – Confusing Client characteristics: – Poor health literacy – Distrust of health services	**Confident clinicians**	Clinician knowledge of health system and of local services Client navigating the health system Wrap around care with appropriate referrals Improved client outcomes Planning for the future

**Table 4:** CMO configuration for Service Collaboration.

Characteristic Of Programme	Context	Mechanism	Outcome

Service collaboration	Health system characteristics: – Siloed health system – Resistant to cross-service collaboration – Lack of clarity about sharing of client information Systemic barriers: – Socio-economic determinants of health – historical perceptions of health services – Poor health literacy – Geographical isolation from services Programme characteristics: – Complexity of HHAN programme making it difficult to explain to other services Client characteristics: – Vulnerability	**Shared provider decision-making**	Recognition of established referral pathways Recognition of benefits of integrated care Motivation to collaborate Identification of collaboration opportunities Informal and formal communication Breakdown of silos Collaboration between services Recognition by service partners Utilising appropriate services Acknowledgement and acceptance of HHAN Foundations for integration

### Overarching mechanisms

**Figure 2:**
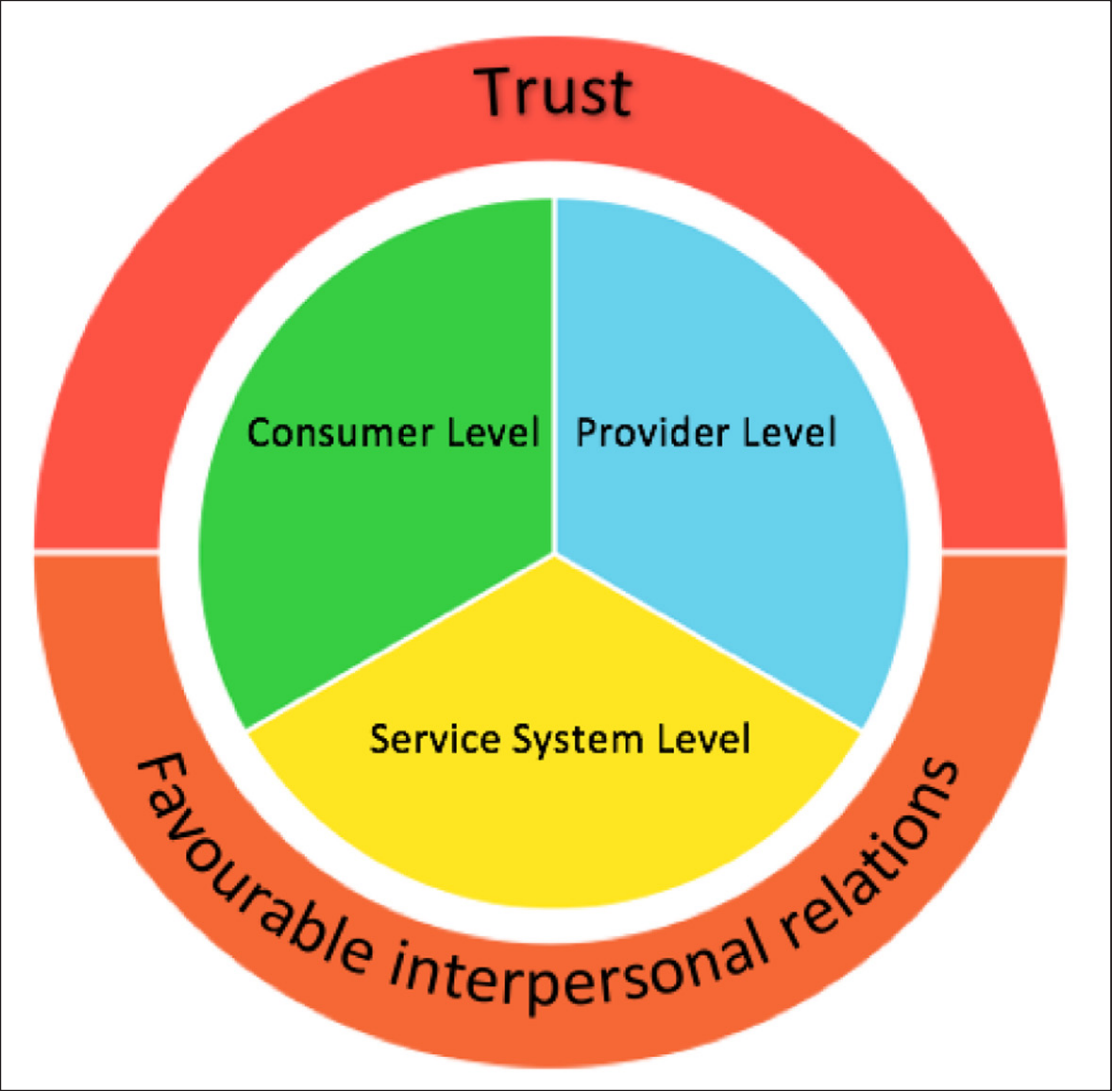
Overarching mechanisms present at all levels of the HHAN logic model.

The two overarching relational mechanisms were present at every level required to facilitate engagement with vulnerable clients. These are therefore presented separately and not incorporated into the CMO configurations (see Figure 2).

The mechanism of “trust” between service-providers and clients was abstracted as a mechanism from all participants’ interviews as the overall factor necessary for engagement in the HHAN model of care and consequently further services. Contextually, some clients expressed distrust in health and social service systems and providers, many due to previous negative experiences with welfare interventions. Such distrust and past experiences shaped how they made sense of subsequent involvement with health services and often resulted in an unwillingness to engage in care. Facilitators of trust in such a context were found to be service-providers responding to the need and priorities of the clients, as these were identified by the clients. This included service-providers offering practical and immediate solutions to clients concerns hereby showcasing their usefulness and engagement. The establishment of an honest, equal relationship was found to activate feelings of trust and assisted in producing positive client outcomes. The below quotes from clients illustrate this dynamic:

“I like real people and they were real people. I like people that don’t sit on the side-lines … they got in my mess you know … I knew they were there for me, but I also had to do things on my own, and where I lacked, that’s where […] came in.” (Client)

“They don’t come to our families with an authoritative attitude, they’re relaxed, they speak in a normal conversation, not ‘I’m above you’, which is important for our families.” (Client)

Favourable inter-personal relations between service-providers and clients emerged as an important relational mechanism. Clients noted that their engagement in the programme was dependent on interpersonal attributes of their clinician, stating they valued and responded to their non-judgmental, supportive, efficient and empathetic nature. This is exemplified by this client statement:

“… you don’t have to worry too much about how you’re seeming to them, you can just be yourself, be honest, and you know they will be honest with you and try and help you in the best way they can.” (Client)

Stakeholders agreed that the open, engaging personalities of clinicians, combined with their extensive knowledge of the health system, facilitated successfully engaging with clients, whilst making them easy to work with at a professional level. As this stakeholder comments

“*It is their personalities, but also being able to navigate the whole system*” (stakeholder).

### Consumer Level

#### Whole of family care

A whole of family care approach emerged as a central mechanism enhancing client acceptability of the HHAN intervention. Whole of family care involves clinicians focusing not on an individual client, but rather on the family as a unit involving adults, children and in many cases extended family members such as grandparents and other family members. The CMO configuration coinciding with the HHAN logic model at the clinical care and consumer level is outlined below (see Table 2).

Contextually, clients experienced a mixture of vulnerabilities and contextual circumstances. Vulnerability in this context comprised of varying structural, social and personal factors, such as financial stresses, housing issues, intergenerational trauma, limited social support, health conditions and challenging social and family experiences such as disconnection from family, grief and loss, domestic violence and histories of children being removed from care. Studies show that intergenerational trauma, including the inheritance of disadvantage from parent to child, may be aided by family-centred care [35,36]. Such interventions look beyond the individual client and target the interrelated dynamics involved in improving the well-being of the whole family. Mechanisms in support of this mode of intervention involve culturally-appropriate, trauma-informed care, emphasis on rapport building, and shared decision-making between clinicians and family members. Clients appreciated the providers taking time to understand their family dynamic and involving all family members as active participants in deciding and prioritising elements targeted for intervention. In the context of disadvantaged families and histories of trauma, the intervention element with ‘shared decision-making’ enabled the activation of mechanisms such as independence and self-determination, important elements for clients seeking to rebuild control over their lives. Additionally, clinicians’ inclusiveness increased clients’ social support and ability to change their circumstances.

“I loved the fact that she took interest, that she took interest in all of us. …they come out to you and they make that time and effort, it gives you this thing to keep on striving … especially when referring you to the right services that are required.” (Client)

Stakeholders echoed the opinions of clients, agreeing that whole of family care was a vital mechanism for gaining client trust as well as trust within the community.

“If you look at their list, you can connect most of them up. HHAN is not just helping one household, they are helping an entire community because there are the cousins, the aunties and uncles … it’s creating this standardised trust”. (Stakeholder)

The mechanisms that operated within the whole of family approach in this context resulted in clients feeling supported, and increased acceptance of HHAN within the extended family networks and the wider community.

### Provider Level

Four provider level mechanisms were identified within the HHAN logic model: accessibility, accompaniment, clinician autonomy, and navigation of the health system (see Table 3). Analysis of the contexts and mechanisms which operate at the provider level highlighted both beneficial and potentially harmful outcomes.

#### Accessibility

The context of accessibility involves client, programme and health system characteristics. Markers of vulnerability such as those tied to structural disadvantage, limited social capital and mental health issues combined with distrust in the health system are present at the client level. Systemically, the inflexibility of the health care system provides the backdrop for the necessity of care coordination for vulnerable families.

Clients highlighted the enabling service-provider characteristics such as workers being approachable, persistent and generally available as mechanisms that aided their continued engagement in the programme. Accessibility was improved by offering various modes of communication, as clients cited appreciating being able to contact their worker via text, call or email. Additionally, HHAN staff provided both home visiting and a place-based service, which greatly improved the flexibility of the service. When asked directly if she would have left home to visit her service-provider, one client stated:

“At the first earlier stages no, I don’t think I would have. I mean I just didn’t want to see anybody, but she did call, she did text, she did messages, emails and came around to see me, ask how I was doing … by coming to my home and saying hi, how is everybody, everyone chilled out a bit.” (Client)

Stakeholders agreed that such programme flexibility ensured that staff could reach families who may otherwise have gone unnoticed by the health system. One service provider stated:

“Every service has their scope and we’re basically saying … we’ll take on these families with children up to 17 and identify all these broad issues and get as many services as we can for the best interests of the child. And … that’s unique, I haven’t seen that anywhere.” (service provider).

Additionally, the accessibility of the clinicians was believed to stimulate service engagement beyond HHAN services.

#### Accompaniment

Accompaniment describes the initial ‘hand-holding’ period required where staff provided supplementary care and support to clients to enable service engagement. Contextual factors facilitating this mechanism exist both at the client level, namely high and complex needs and previous disconnection from health services, as well as at the service level, in particular, the absence of strict programme protocols. Clinicians’ ability to be adaptable to clients’ fluctuating intensity of need were facilitated by service-level mechanisms such as the absence of strict referral criteria, programme outcomes and set timeframes for how long clients could be engaged in the service. This flexibility allowed clinicians to respond resourcefully and immediately to a variety of needs of all family members and connect them in with appropriate local services. Increasing clients’ knowledge of, and access to, local resources available to them greatly improved clients’ ability to take care of themselves and their family. As one client mentioned:

“She’s given me some guidance, I’m familiar with where I should go now and what needs to happen … I’m comfortable doing so.” (Client)

Relational mechanisms such as clinician persistence and reliability, combined with client motivation to better their circumstances, often resulted in feelings of independence and self-awareness for clients. However, the risk of dependency was identified by both clients and providers, as clients could become overly reliant on their service-providers.

“Bit by bit I’m getting my life back together … but I am finding the thought of her not being with us difficult, because I’m becoming reliant on her.” (Client)

The mechanism of flexibility in instances where there was role ambiguity or limited role clarity for staff, raised issues for clinicians. Clinicians voiced concern over crossing the boundaries from providing care coordination into providing case management, generally a more time-intensive and involved role. Staff expressed that a gradual reversal into a care coordination role, after a period of providing case management support, could be complicated by the severe disadvantage of many clients.

“You’re trying to develop that sort of therapeutic relationship. That takes time … within that sort of initial spike you definitely case-manage, until you can find a way to identify those services that can come in and take over your role. Or unfortunately, if there are no services, you become that person.” (Service-provider)

As such, identical contextual elements and mechanisms led to a third outcome: the emergence of a case management model of care. The overarching structural context for this process is the insufficient social and health support available for disadvantaged families. Additionally, the previous distrust in and disconnection from health services experienced by many clients led to a reluctance to establish connections with mutable service-providers, once a trusting relationship was developed with a key worker. In such cases, staff habitually felt unwilling or unable to resign the case managing role.

#### Clinician autonomy

To be able to navigate the flexible programme design and the intensified relational demands of working with disadvantaged clients with complex needs, clinicians needed to be both experienced and able to work independently. Service-providers were required to be adaptable due to the diversity of clients and family characteristics such as age range, social and health issues and cultural backgrounds. Habitually, clinicians were required to work outside of what was considered the scope of their professional practice (i.e. those tied to the social work or clinical nurse consultatn job roles). The clinicians’ job motivation, personal flexibility, problem-solving ability and confidence in the benefits of HHAN were facilitating mechanisms for this mode of intervention. Below is a quote from a stakeholder discussing the multi-faceted nature of the clinician role.

“…you have an understanding with a worker about what you’re trying to achieve, they can link you in and give you information about different things … Healthy Homes is a more rounded thing where they want everything to be balanced and working well, instead of just looking at one side of things.” (Stakeholder)

Identical context-mechanism combinations also resulted in negative programme outcomes. Service-providers acknowledged that whilst the role flexibility provided much needed autonomy to deliver truly client-centred care, service delivery, at times, became dependent on the personality and unique skill-set of clinicians. Moreover, clinicians described how the personality-driven nature of the clinician role created an enormous sense of responsibility for clients’ well-being. Such work stressors were felt to increase the risk of staff burnout.

“Clients divulge some incredibly personal and quite disturbing histories at times … and I think that’s a real challenge within this job. It’s that there isn’t a client who isn’t challenging, so there’s not down time. We can’t fix our clients, and I think that’s a different way of thinking compared to nursing or medicine … with HHAN, you don’t leave.” (Service-provider)

Such a context of complicated client needs, an emphasis on creating trusting therapeutic client-clinician relationships and the danger of role ambiguity, made it challenging for clinicians to assert healthy boundaries with their clients. In light of the challenging work performed, workers occasionally struggled to reconcile working empathically and professionally with vulnerable families. Work intensification is a significant problem for workers dealing with high-need clients [37]. Mechanisms built into the logic model to prevent work intensification and staff burnout included the clinicians being personally responsible for their own caseloads, as opposed to having to work to a set number of clients. This finding highlights the importance of providing additional institutional support in the form of de-briefing opportunities and professional supervision when working with vulnerable groups in such a flexible service delivery model.

#### Navigation of the health system

Another vital element of the programme highlighted by clients and staff related to assisting clients in navigating the health system. Within the context of a confusing health system, poor health literacy and distrust in welfare services, many clients reported benefitting from receiving such assistance:

“It’s her knowledge … and if there’s something she can’t assist with, she’ll say I’ll look into it and get back to you. She gives you the information and says would you like me to advocate for you or can you manage? … I guess she uses whatever resources she has, but it’s her knowledge.” (Client)

Being able to navigate the health system, initially with assistance and eventually on their own, was described as an empowering experience by many clients. Clients described how being able to care for and seek support for themselves and their family had made additional resources available within the family unit and opened possibilities of receiving further support into the future. As this client reports:

“It has given me opportunities to do things I didn’t think I was going to do … now I have my learners license … I am pregnant and having another child, I’m going to have a home nurse come out and check up on the baby.” (Client)

Several mechanisms were established to assist clients in navigating the health system. The context of this mechanism is similar to the context reported under the themes accompaniment and client autonomy, as both flexibility and the gradual pulling back of support to facilitate client self-direction and autonomy helped produce good outcomes.

### Service System Level

Service collaboration is the major mode of intervention for the service system level of HHAN, outlined by the CMO configurations illustrated in Table 4. Whilst the clients were largely unaware of the service-providers’ work to integrate the services around them, clinicians and stakeholders believed it to be a major part of the care coordinator role, mirroring the initial HHAN logic model at the service system level (see Table 1).

#### Service collaboration

The majority of participants spoke about the importance of services and practitioners working together in a coordinated and client-focussed way to respond appropriately to the often complex support needs of clients. Effective inter- and intra- service cooperation is influenced by multiple factors such as coordination, partnership models, preparedness to share power and information, challenges to professional identity, competing goals and agendas across services, and lack of reconciliation of different ways of working [8]. Stakeholders in this study highlighted similar systemic, service and practitioner-level barriers preventing effective collaboration such as the fragmented service environment, underdeveloped pathways for intra- and interagency collaboration, competing priorities of services, widespread service and clinician-level resistance, and limited procedures for information sharing across services and sectors (listed in Table 4). The complexity of the HHAN programme, and difficulties of other services in appreciating where a care coordination service might fit within the suite of other services, further complicated service collaboration.

Stakeholders believed the readiness of HHAN clinicians to work with other services and their recognition of existing service pathways operated as mechanisms in successful collaboration. A general recognition that working together would enable better outcomes for clients was a mechanism facilitating collaboration as the quote below illustrates:

“It’s been about the relationships across the table … that willingness to form relationships and respect for other services I think is what pushes this project along.” (Stakeholders)

Clinicians echoed this belief in collaboration and spoke of the informal and formal communication that enabled referral pathways and service collaboration. The creation of trusting relationships between service-providers and services were a facilitating mechanism in this regard.

“There is no way I could operate by myself in this job. … I need the other services and I think it’d be foolish to not work really hard on trusting and having those professional relationships with other services. … once you get all the services on board, it seems to be a lot easier.” (Service-provider)

Whilst stakeholders believe in the value of integration, interviews also demonstrated that a historic fragmentation of health services and insufficient processes enabling staff to work across silos had led to a delay in service integration. Furthermore, practitioners stated that whilst collaboration may be increasingly evident at the service-provider level, at a systemic level, many barriers remained.

“There’s definite recognition, acknowledgement, at least verbally. That hasn’t always translated into systems practice change.” (Service-provider)

The goal of intra- and inter-sectoral collaboration was often compromised by a wide-spread fragmentation of services and services operating in silos, a finding well described in the literature [38,39]. Ultimately such barriers had the potential to affect client outcomes.

## Discussion

This study has discussed a range of contextual factors and mechanisms relating to a defined aspect of the HHAN logic model, namely the care coordination aspect, and how these can combine and either produce positive or negative outcomes for clients and the health system. In this way, data highlighted structural, institutional and individual-level drivers of good client outcomes and the organisational context in which such outcomes are embedded.

Overall, trust and favourable interpersonal relations were found to be overarching mechanisms, under which secondary modes of intervention at client, provider and system levels emerged. The centrality of trust and favourable interpersonal relations as enablers of service engagement and provider motivation to collaborate has been confirmed in recent literature [21,40]. The findings from this study further develop such findings by suggesting that interpersonal dynamics should be seen as an organisational strength to be actively nurtured. Consequently, service design should focus on fostering the creation of positive relationships at all levels to ensure good client outcomes. This has implications for job design, recruitment, workforce capability development, client-ratios and service to service communication pathways, and should be factored into the timeline for program implementation.

Rapport building with all family members, acknowledgement of family dynamics and past experiences of vulnerable families, are mechanisms that have been found to promote positive outcomes such as client independence, improved outlook and community acceptance of the programme [41]. Under contexts and conditions where vulnerable families experienced distrust of past health services, a whole of family care approach can allow clients to feel supported in their community. When implementing such a program, it is therefore important to consider the workforce delivering the program and ensure that service providers and partners are sufficiently placed to address the unique needs of all family members across the lifespan. Effort must be made to engage stakeholders who may not traditionally contribute to the delivery of a “child and family” program, ensuring that referral pathways are accessible for these families.

Access to appropriate care and support services can be difficult for disadvantaged families with complex needs, and structural, social and economic characteristics can complicate this further [9]. A major programme element, accessibility, was achieved through the availability of place-based and home visit services. This contextual setting, combined with flexible means through which clients could contact clinicians, made it easier for clients to engage with services.

Two CMO outcomes emerged under the theme accompaniment; client independence and client reliance. Separate CMO configurations could not be distinguished for each outcome, exemplifying the complexity of HHAN as a multifaceted programme. Future analysis of HHAN should focus on defining a specific causality for each response to allow the opportunity for the programme to be modified. The risk of the case management model emerging should also be reviewed. Whilst research recommends a combination of accompaniment and navigation, this study suggests it must be done with caution due to the risk of HHAN siloing its own service [9].

Clinician autonomy is comprised of unique contextual elements, including the clients’ range in demographics and programme flexibility. Allowing a highly skilled clinician the freedom to make shared decisions with clients resulted in increased service engagement and an improved sense of independence for clients, as also shown in other studies [40]. Thus, programmes operating in a similar context should consider the importance of employing senior staff that can adapt within a programme of flexible design. However, it must be noted that this high level of autonomy also puts the clinicians at risk of burnout, and relevant and effective professional learning and support systems should be considered in programme design.

The fourth element at a provider level – service navigation – highlighted the impact the confusing nature of the health system can have on clients. The range of outcomes generated within this programme element demonstrates that knowledge transfer achieved from linking an experienced clinician with a client of limited health literacy can go beyond creating wrap-around care for clients. More so, service navigation allowed clients the ability to self-navigate the health system, improving confidence in their own capabilities, and improving their likelihood of making attainable plans for their future. These changes in clients’ sense of self highlight the significant impact isolation from the health sector can have [42].

Service collaboration emerged as a major mode of intervention at the service system level, as also reflected in the programme theory. The breakdown of silos and the collaboration between services mentioned by stakeholders confirm the value of the HHAN clinicians focussing their time on relationship building with services, as well as with clients. Despite this, interview data also indicates that whilst there is service collaboration occurring, there is not yet widespread service integration. Both mechanisms that promote and hinder service collaboration are present, highlighting the need to pay further attention to the complexities of service collaboration within a health care context. Examining the strengths and barriers to service collaboration at all levels, utilising patient stories to demonstrate the impact this has on families, may be a strategy to improve stakeholder engagement and drive further collaboration. Further, working at multiple levels at the same time and maximising opportunities for partnership, shared learning and knowledge transfer through multiagency shared case work may aid in transferring learnings from the service level to the system level, and assist with the transition from service collaboration to service integration.

### Strengths and limitations

A strength of this study is the realist design, which provided a useful and thorough methodology for evaluation. The principle of theorising the interviews to determine CMO configurations allowed for a deeper understanding between context, mechanisms and outcomes of HHAN care coordination, and therefore an insight into the complexities of the relationship between intervention elements and client, provider, and service outcomes [25].

The study, however, has several limitations. The exact CMO configuration for each major element was difficult to define. Whilst several analytical methods were employed, such as theory gleaning, refining and consolidating [31], immersion crystallisation as well as extensive team discussion, the risk of misattribution of causality remains. Different causal processes could produce the same result, just as the same intervention could trigger a range of outcomes. The interviews for this evaluation were undertaken between December 2015 and September 2017, over which time the programme was still developing. Whilst no major disparities were evident between interviews, this time frame must be noted. This study may be subject to participant bias, as clients who agreed to be interviewed may have strong opinions for or against HHAN delivery. Furthermore, the interviews were undertaken for an evaluation of the entire HHAN programme, and therefore included questions not relevant to care coordination specifically.

## Conclusions

This paper has discussed the value of combining care coordination at a family level, with service integration at a systemic level. Service collaboration is necessary for the breakdown of silos, however this is difficult and systemic and service level resistance to collaboration remains and can impede effective service integration. Through determining the generative causation for each mode of intervention, this study allows the negative outcomes to be addressed and the positive outcomes to be understood, providing a key platform for future care coordination programs to develop and address system inadequacies.

The findings from this evaluation have important implications for future developments of the HHAN programme, as well as similar integrated care programmes. The central role of trust and favourable interpersonal relations as underlying mechanisms impacting upon all facets of programme delivery was a strong finding in the evaluation. Core elements of care coordination for vulnerable families as highlighted by clients, service-providers and stakeholders included whole of family care, accessibility and service navigation. A flexible programme model was required to facilitate the emergence of a care coordination model of care. Flexible programme design and clinician autonomy created positive client outcomes, however additional institutional support needed to be put in place to avoid role ambiguity and staff burnout. Service collaboration is necessary for the breakdown of silos, however, systemic and service level resistance to collaboration remains, impeding on effective service integration.

## References

[1] Taplin S, Mattick RP. Mothers in methadone treatment and their involvement with the child protection system: A replication and extension study. Child Abuse and Neglect, 2013; 37(8): 500–510. DOI: 10.1016/j.chiabu.2013.01.003 23428166

[2] Price-Robertson R. What is community disadvantage. Understanding the issues, overcoming the problem, 2011: 10.

[3] Saunders P, Naidoo Y, Griffiths M. Towards New Indicators of disadvantage: Deprivation and Social Exclusion in Australia. Social Policy Research Centre, UNSW 2007 DOI: 10.1002/j.1839-4655.2008.tb00097.x

[4] Vinson T, Rawsthorne M, Beavis A, Ericson M. Dropping off the Edge – Persistent communal disadvantage in Australia Australia: Jesuit Social Services/Catholic Social Services Australia; 2015.

[5] The Royal Children’s Hospital Melbourne. Place-based initiatives transforming communities: proceedings from the place-based approaches roundtable Melbourne, Australia: The Royal Children’s Hospital Melbourne; 2012.

[6] Ward P, Coates A. ‘We shed tears, but there is no one there to wipe them up for us’: narratives of (mis)trust in a materially deprived community. Health: An Interdisciplinary Journal for the Social Study of Health, Illness and Medicine, 2006; 10(3): 283–301. DOI: 10.1177/1363459306064481 16775016

[7] McLachlan R, Gilfillan G, Gordon J. Deep and Persistent Disadvantage in Australia Canberra: Productivity Commission Staff Working Paper; 2013.

[8] Grace R. Hard-to-reach or not reaching far enough? Supporting vulnerable families through a coordinated care approach A review of the literature to support the Healthy Homes and Neighborhoods Project. Sydney, Australia: Children and Families Research Centre, Macquarie University; 2015.

[9] OECD. Integrating social services for vulnerable groups: Bridging sectors for better service delivery Paris: OECD Publishing; 2015.

[10] World Health Organisation: Social Determinants of Health. http://www.who.int/social_determinants/sdh_definition/en/; 2017.

[11] What is integrated care? [https://www.health.nsw.gov.au/integratedcare/Pages/what-is-integrated-care.aspx].

[12] Alexander K, Brown M, Halim S, Hendry A, Eastwood J. Child and family health indicators report: Inner west Sydney 2013 Sydney, Australia: Community Paediatrics, Sydney Local Health District; 2015.

[13] Eastwood J. Designing initiatives for vulnerable families: from theory to design in Sydney, Australia. International Journal of Integrated Care, 2017; 17(5): 1–8. DOI: 10.5334/ijic.3540 PMC6659580 31367208

[14] Grace, R. Hard-to-reach or not reaching enough? Supporting vulnerable families through a coordinated care approach. A review of the literature to support Healthy Homes and Neighbourhoods Project In: Sydney: Children and Families Research Centre, Macquarie University; 2015.

[15] NSW Health. Integrated Care Key Initiatives. [https://www.health.nsw.gov.au/integratedcare/Pages/key-initiatives.aspx].

[16] Schultz EM, McDonald KM. What is care coordination? International Journal of Integrated Care, 2014; 17(1–2): 5–24. DOI: 10.1177/2053435414540615

[17] Sydney Local Health District. Inner West Sydney Health Homes and Neighbourhoods: 2015–2016 Annual Report Sydney, Australia: http://www.slhd.nsw.gov.au/pdfs/HealthyHomesAnnualReport.pdf; 2016.

[18] Eastwood J, Shaw M, Garg P, De Souza D, Tyler I, Dean L, McSween M, Moore M. Designing an Integrated Care Initiative for Vulnerable Families: Operationalisation of realist causal and programme theory, Sydney Australia. International Journal of Integrated Care, 2019; 19(3): 10 DOI: 10.5334/ijic.3980 PMC6659766 31367209

[19] Eastwood J, De Souza D, Shaw M, Garg P, Woolfenden S, Tyler I, Kemp L. Designing initiatives for vulnerable families: from theory to design in Sydney, Australia. International Journal of Integrated Care, 2019; 19(3): 10 DOI: 10.5334/ijic.3980 PMC6659580 31367208

[20] Nelson A. Unequal treatment: Confronting racial and ethnic disparities in health care. Journal of the National Medical Association, 2002; 94(8): 666–668. 12152921 PMC2594273

[21] Olley H, Psaila K, Fowler C, Kruske S, Homer C, Schmied V. ‘Being the bridge and the beacon’: a qualitative study of the characteristics and functions of the liaison role in child and family health services in Australia. Journal of Clinical Nursing, 2016; 26(1–2): 91–102. DOI: 10.1111/jocn.13373 27647750

[22] Craig P, Dieppe P, Macintyre S, Michie S, Nazareth I, Petticrew M. Developing and evaluating complex interventions: New guidance. BMJ (Clinical Research Ed), 2008; 337(a1655). DOI: 10.1136/bmj.a1655 PMC2769032 18824488

[23] Eastwood J, Kemp L, Jalaludin B. “Being Alone and Expectations Lost”: A Realist Theory of Neighborhood Context, Stress, Depression, and the Developmental Origins of Health and Disease. Sage OPen, 2018; 8(1): 2158244018763004 DOI: 10.1177/2158244018763004

[24] Pawson R, Tilley N. Realist evaluation London: SAGE; 1997.

[25] Wong G, Westhorp R, Manzano A, Greenhalgh J, Jagosh J, Greenhalgh T. RAMESES II reporting standards for reliast evaluations. BMC Medicine, 2016; 14(96). DOI: 10.1186/s12916-016-0643-1 PMC4920991 27342217

[26] UK Medical Research Council. Developing andevaluating complex interventions London: UK Medical Research Council; 2006.

[27] Eastwood J, Kemp L, Garg P, Tyler I, De Douza D. A Critical Realist Translational Social Epidemiology Protocol for Concretising and Contextualising “Theory of Neighbourhood Context, Stress, Depression, and the Developmental Origins of Health and Disease (DOHD)”, Sydney Australia. International Journal of Integrated Care 2019, 19(3). DOI: 10.5334/ijic.3962 PMC6659581 31367207

[28] Eastwood J, Woolfenden S, Miller E, Shaw M, Garg P, Liu H, De Souza D, Ettema R. Implementation, mechanisms of effect and context of an integrated care intervention for vulnerable families in Central Sydney Australia: A research and evaluation protocol. International Journal of Integrated Care, 2019, 19(3): 11 DOI: 10.5334/ijic.4217 PMC6659760 31367210

[29] Layder D. New strategies in social research: An introduction and guide Cambridge: Polity Press; 1993.

[30] Patton MQ. Qualitative research and evaluation methods 3rd Thousand Oaks, CA: Sage Publications; 2002.

[31] Manzano A. The craft of interviewing in realist evaluation. Evaluation 2016, 22(3): 342–360. DOI: 10.1177/1356389016638615

[32] Bhaskar R. The possibility of naturalism: A philosophical critique of the contemporary human sciences (3rd ed.). Florence, USA: Routledge; 1998.

[33] Thornberg R, Charmaz K. The SAGE handbook of qualitative data analysis London: SAGE; 2013.

[34] Brazley P. Qualitative Data Analysis: Practical strategies (J. Seaman Ed.). London: SAGE; 2013.

[35] Berger LM, Font SA. The Role of the Family and Family-Centered Programs and Policies. The Future of Children, 2015; 25(1). DOI: 10.1353/foc.2015.0007 PMC6342196 30679897

[36] Cheng TL, Johnson SB, Goodman E. Breaking the Intergenerational Cycle of Disadvantage: The Three Generation Approach. Pediatrics, 2016; 137(6). DOI: 10.1542/peds.2015-2467 PMC4894258 27244844

[37] Henderson J, Willis E, Walter B, Toffoli L. Community mental health nursing: Keeping pace with care delivery? International Journal of Mental Health Nursing, 2008; 17(3): 162–170. DOI: 10.1111/j.1447-0349.2008.00528.x 18460077

[38] Simpson SH. Of silos and systems: the issue of regionalizing health care. The Canadian journal of hospital pharmacy, 2011; 64(4): 237 DOI: 10.4212/cjhp.v64i4.1033 22479064 PMC3161795

[39] O’Shaughnessy CV. Breaking Down Silos of Care: Integration of Social Support Services with Health Care Delivery. National Health Policy Forum, 2012.

[40] Stephenson M, Campbell J, Lisy K, Chu WH, Aromataris E. Providing integrated care: experiences of healthcare providers http://www.health.nsw.gov.au/wohp/Documents/provider-experience-lit-review.pdf The Joanna Briggs Institute; 2015.

[41] Woodruff J, O’Brien J. Children’s and family services working together. Australian Journal of Early Childhood, 2005; 30(1): 49–57. DOI: 10.1177/183693910503000109

[42] Drummond J, Schnirer L, So S, Mayan M, Williamson DL, Bisanz J, Fassbender K, Wieve N. The protocol for the Families First Edmonton trial (FFE): a randomized community-based trial to compare four service integration approaches for families with low-income. BMC Health Services Research, 2014; 14(223): 1–12. DOI: 10.1186/1472-6963-14-223 24885729 PMC4060625

